# Predictors of poor functional outcomes and mortality in patients with hip fracture: a systematic review

**DOI:** 10.1186/s12891-019-2950-0

**Published:** 2019-11-27

**Authors:** Bang Yu Xu, Shi Yan, Lian Leng Low, Farhad Fakhrudin Vasanwala, Sher Guan Low

**Affiliations:** 1 Department of Post Acute and Continuity Care, SingHealth Community Hospital – Sengkang, 1 Anchorvale St, Singapore, 544835 Singapore; 2 Duke-NUS Medical School, 8 College Road, Singapore, 169857 Singapore; 3 Department of Family Med & Continuing Care, Singapore General Hospital, Outram Rd, Singapore, 169608 Singapore

**Keywords:** Hip fractures, Predictors, Functional outcomes, Mortality

## Abstract

**Background:**

Hip fracture is an important and prevalent medical condition associated with adverse outcomes. The aim of this article is to systematically review and summarise the predictors of poor functional outcomes and mortality for patients with hip fractures.

**Methods:**

We conducted a systemic literature search using PubMed, EMBASE and Cochrane Library. We included English peer-reviewed cohort studies that examined predictors of poor functional outcomes (such as independence in Activities of Daily Living) and mortality for patients with hip fracture published in the past 15 years (from 1 Jan 2004 up to 30 May 2019). Two independent researchers evaluated the articles for eligibility. Consensus on the eligibility was sought and a third researcher was involved if there was disagreement. A standardised form was used to extract relevant data. The Newcastle-Ottawa Scale (NOS) was used to assess the quality of the included studies.

**Results:**

We retrieved 4339 and included 81 articles. We identified two emerging predictors of poor functional outcomes and mortality for patients with hip fractures: low hand grip strength and frailty in line with an emerging concept of “physical performance”. The predictors identified in this systematic review can be grouped into 1) medical factors, such as presence of co-morbidities, high American Society of Anesthesiologists (ASA) grade, sarcopenia, 2) surgical factors including delay in operation (e.g. > 48 h), type of fracture s, 3) socio-economic factors which include age, gender, ethnicity, and 4) system factors including lower case-volume centers.

**Conclusions:**

This systematic review identified multiple significant predictors of poor functional outcomes and mortality, with the hand grip strength and frailty being important emerging predictors in the most recent literature. These predictors would further inform healthcare providers of their patients’ health status and allow for early intervention for modifiable predictors.

## Introduction

Hip fracture is an important medical condition associated with adverse outcomes, including mortality [1]. The incidence of hip fractures is expected to increase due to ageing populations worldwide - there were 1.6 million hip fractures worldwide in year 2000 and this number is expected to increase to 4.5–6.3 million by 2050 according to International Osteoporosis Foundation [1, 2]. One-year mortality rate for patients with hip fracture was reported to be up to 20–24% and the mortality risk may persist beyond 5 years [3, 4]. As for functional outcomes, it was reported that 40% of hip fracture patients were unable to walk independently, 60% required assistance, and 33% were totally dependent or in a nursing home 1 year after hip fracture [3, 5, 6]. With increasing incidence and associated poor clinical outcomes, the impact of hip fractures on the healthcare system is significant.

Previous studies reported various predictors of adverse clinical outcomes for patients with hip fractures. A recent systematic review identified several predictors of mortality up to 12 months including cognitive impairment, age > 85 years and pre-fracture mobility [7]. However, it did not examine other important clinical outcomes other than mortality, especially functional ability. “Developing and maintaining the functional ability that enables well-being” has been the new vision of healthy ageing by World Health Organization [8]. Information about patient’s functional outcome is especially important given that the rapid ageing populations worldwide have resulted in increasing attention from researchers and policy makers to ageing related syndromes affecting patients’ functioning such as sarcopenia and frailty [9, 10].

It is well recognized that muscle function and physical performance are important clinical information that are relevant to patients’ functioning [11, 12]. A recent work by European Society for Clinical and Economic Aspects of Osteoporosis and Osteoarthritis (ESCEO) working group on frailty and sarcopenia reviewed large number of approaches to measure muscle function and physical performance and recommended the use of grip strength to measure muscle strength and the use of 4-m gait speed or the Short Physical Performance Battery test to measure physical performance in daily practice [11]. In fact, grip strength has been the measure of choice for the assessment of overall muscle strength for the diagnosis of sarcopenia and frailty, as it has standardized, validated, easy to use protocols [13–15]. Given the rapid development and global emphasis on functional ability of the elderly, it is imperative to conduct an updated review on patients with hip fractures to include functional outcomes.

This review aims to summarize the existing literature on predictors of poor functional outcomes and mortality for patients with hip fractures. This would provide the latest evidence-based information that would assist healthcare providers to target modifiable predictors in order to reduce the incidence of poor outcomes.

## Methods

### Data sources and searches

We performed a systematic literature search for published literature in the past 15 years (from 1 Jan 2004 up to 30 May 2019) in three databases PubMed, EMBASE and Cochrane Library according to the Preferred Reporting Items for Systematic review and Meta-Analysis (PRISMA®) checklist. We chose to review the articles in the recent 15 years because by focusing on more recent data, we can summarize the evidence relevant to today’s medical practice. Hand search was also performed based on the references from the included studies.

Using the PubMed Advanced Search Builder, the following key search terms were used:

Critical Care Outcomes[Mesh] OR Patient Outcome Assessment[Mesh] OR Outcome Assessment (Health Care)[Mesh] OR Patient Reported Outcome Measures[Mesh] OR Fatal Outcome[Mesh] OR Treatment Outcome[Mesh] AND Hip Fractures[Mesh] AND predict*.

The detailed search strategy for the three databases can be found in Additional File 1.

### Study selection

Two independent researchers evaluated the articles for eligibility through screening of the title and abstract first, followed by full text. Consensus on the eligibility of the articles was sought and the third researcher was involved if there was disagreement.

We included English peer-reviewed cohort studies that examined poor functional outcomes and mortality for patients with hip fracture published in the past 15 years (from 1 Jan 2004 up to 30 May 2019). Exclusion criteria were studies with inappropriate format (e.g. audit, self-administered survey, cross-sectional studies, systematic reviews, randomized controlled trials, case reports, and poster abstracts), and non-English articles.

### Quality assessment

The Newcastle-Ottawa Scale (NOS) was used to assess the quality of cohort studies [16],

## Results

**Fig. 1:**
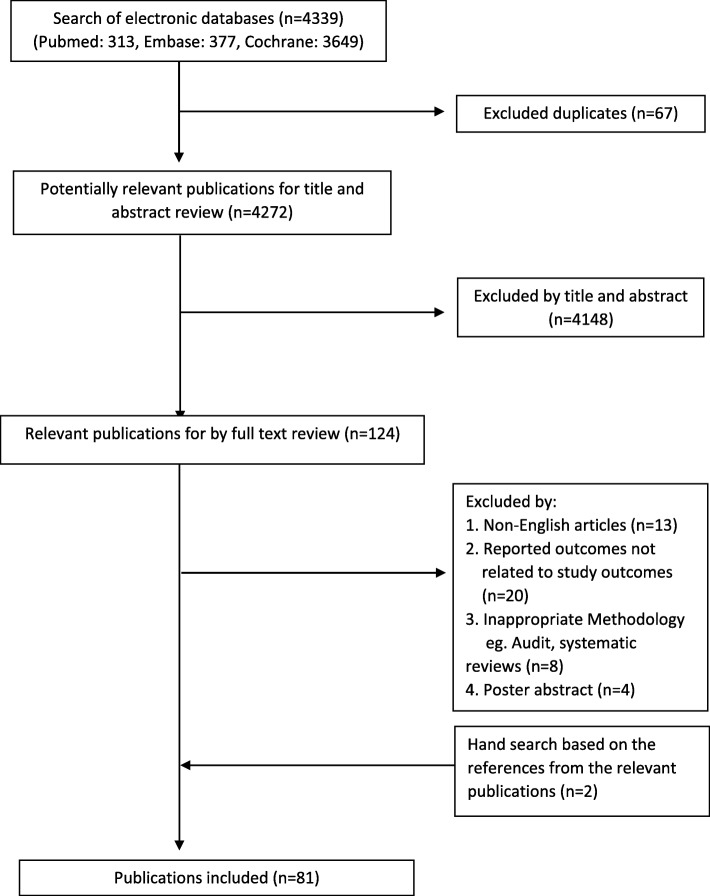
Flowchart of review process

As shown in Fig. 1, 4339 articles were retrieved from the initial search process. One hundred twenty-four articles are potentially relevant for full text review after removing 67 duplicates and 4148 articles by title and abstract. Eighty-one articles were included in this article after full text review further excluded 43 articles. A summary of the included articles is presented in Additional file 1 [17–97]. Predictors of poor functional outcomes and mortality for patients with hip fracture are grouped into medical, surgical, socio-economic and system factors. The excluded articles based on full text review are listed in Additional file 1.

Table 1 showed the predictors of poor functional outcomes. The medical predictors of poor functional outcomes include poor pre-fracture functional status, cognitive impairment, presence of multiple co-morbidities, high ASA grade, low hand grip strength, Body Mass Index (BMI), sarcopenia (as defined by The European Working Group on Sarcopenia in Older People Criteria [98]), frailty, depression, serum albumin and folic acid level, visual impairment, heart failure, hypercholesterolemia, osteoporotic treatment, osteoarthritis, pressure ulcers. The surgical predictors are extra-capsular fractures, delay in surgery for more than 48 h, associated dislocation and non-weight bearing status post-surgery. Older age, male gender, and place of residence are socio-economic predictors of poor functional outcomes. Process of care and length of stay are system predictors of poor functional outcomes.

**Table 1:** Summary of review findings: Predictors of functional outcomes

Factors	Outcome	Frequency of studies reporting association	Studies
Socio-economic Factors			
Age	• Poor outcomes with older age	19/20	[22, 25, 26, 29, 32–34, 36, 38–41, 75, 77, 79, 83, 86, 91, 93]
	• Poor outcomes with age group 80–89 years old	1/20	[90]
Gender	• Female more likely poor outcomes • Male more likely poor outcomes • No difference	2/9 5/9 2/9	[37, 40] [23, 27, 39, 45, 46] [25, 41]
Place of residence	Not living in own home poor outcomes	2/2	[22, 25]
Race/Ethnicity	• Minority race compared to non-Hispanic whites has poor outcomes • Malay compared to non-Malay has poor outcomes	1/2 1/2	[22] [33]
Socioeconomic status	Poor outcomes with poverty	1/1	[22]
Marital status	Poor outcome with no marriage	1/1	[84]
Medical Factors			
Pre-fracture functional status	• Low pre-fracture functional status poor outcomes • High pre-fracture functional status poor outcomes	27/28 1/28	[17, 20, 22, 24, 25, 29, 31–34, 37, 38, 40–42, 44–46, 75, 77–79, 83, 84, 86, 89, 91] [26]
Cognitive impairment	Poor outcomes with cognitive impairment	24/24	[17, 19, 22, 24, 25, 27, 32–34, 37, 40, 41, 43–47, 75–78, 86, 91, 97]
Presence of co-morbidities	Poor outcomes with presence of co-morbidities	8/8	[22, 24, 30, 33, 39, 47, 79, 91]
American Society of Anesthesiologists (ASA)	Poor outcomes with higher ASA scores	7/7	[23, 25, 30, 32, 34, 75, 79]
Hand grip strength	Poor outcomes with low grip strength	4/4	[27, 28, 35, 85]
Body Mass Index (BMI)	Outcomes not related to high BMI	1/1	[42]
Sarcopenia	Poor outcomes with sarcopenia	1/1	[18]
Frailty	Poor outcomes with frailty	1/1	[21]
Depression	Poor outcomes with depression	2/2	[29, 43]
Serum albumin and folic acid level	Poor outcomes with low serum albumin or folic level	1/1	[86]
Visual impairment	Poor outcomes with visual impairment	1/1	[86]
Heart failure	Poor outcomes with heart failure	1/1	[86]
Hypercholesterolaemia	Poor outcomes with the absence of hypercholesterolaemia	1/1	[90]
Osteoporotic treatment	Poor outcomes with absence of osteoporotic treatment	1/1	[94]
Osteoarthritis	Poor outcomes with higher grade of osteoarthritis	1/1	[97]
Pressure ulcers	Poor outcomes with pressure ulcers	1/1	[77]
Surgical Factors			
Fracture type	Poor outcomes with extra-capsular fracture types	5/5	[25, 27–29, 42]
Delay to Surgery	Poor outcomes with delay to surgery > 48 h	3/3	[23, 26, 83]
Weight-bearing status	• Poor outcomes with non-weight bear status post-op • Weight bearing status not associated with outcomes	3/4 1/4	[19, 24, 34] [36]
Associated dislocation	Poor outcomes in patient with fracture and associated dislocation	1/1	[87]
System Factors			
Process of care	Poor outcomes with poor process of care	1/1	[80]
Length of hospital stay	Poor outcomes with longer length of stay	2/2	[19, 29]

Table 2 showed the predictors of mortality. The medical predictors of mortality are presence of multiple co-morbidities, high ASA grade, cognitive impairment, poor pre-fracture functional status, poor functional level at discharge, cardiac diseases, frailty, cancer, renal failure, cerebrovascular accident, diabetes, delirium, malnutrition, and low hemoglobin levels. The surgical predictors of mortality include delay in surgery for more than 48 h, extra-capsular fractures, perioperative fracture and non-operative management of hip fractures. Older age, male gender and being a resident in institutional care homes are socio-economic predictors of mortality. Lower case-volume centers (< 12 cases over 2 years), poor nurse staffing (low ratio of nurses to bed) and inappropriate prescription (medication prescriptions not consistent with clinical guidelines) were system predictors of mortality.

**Table 2:** Summary of review findings: predictors of mortality

Predictor	Outcome	Frequency of studies reporting association	Studies
Socio-economic Factors			
Age	Greater mortality with increasing age	20/20	[48–54, 56, 59, 63, 65–70, 76, 79, 81, 93]
Gender	Males have higher mortality	15/15	[49–51, 53, 56, 57, 59, 63, 66–68, 70, 81, 93, 96]
Institutional care homes residence	Greater mortality in institutional care homes	4/4	[49, 51, 53, 65]
Medical Factors			
Co-morbidities	• Greater mortality with multiple co-morbidities	14/15	[48, 50–54, 56, 59, 66, 68, 72, 79, 82, 88]
	• Greater mortality with less co-morbidities	1/15	[93]
American Society of Anesthesiologists (ASA)	• Greater mortality with higher ASA score • ASA does not predict mortality	8/9 1/9	[49, 52, 62, 63, 68, 70, 72, 88] [71]
Cognitive impairment	Greater mortality with cognitive impairment	9/9	[48, 49, 54, 65, 69, 70, 79, 93, 6]
Pre-fracture functional status	Greater mortality with poor pre-fracture functional status	7/7	[49, 65, 70, 78, 79, 83, 93]
Functional level at discharge	Greater mortality with poor functional status at discharge	3/3	[48, 70, 75]
Cardiac diseases	Greater mortality with cardiac diseases	4/4	[53, 57, 66, 81]
Frailty	Greater mortality with frailty	2/2	[52, 58]
Cancer	Greater mortality with cancer	2/2	[53, 76]
Renal failure	Greater mortality with renal failure	2/2	[53, 57]
Cerebrovascular accident	Greater mortality with cerebrovascular accident	2/2	[53, 81]
Delirium	Greater mortality with delirium	1/1	[93]
Diabetes mellitus	Greater mortality with diabetes mellitus	1/1	[67]
Malnutrition	Greater mortality with malnutrition	1/1	[49]
Hemoglobin levels	Greater mortality with lower hemoglobin level	1/1	[95]
Surgical Factors			
Delay in operation	• Greater mortality with delay in surgery • No difference in mortality based on time of day the surgery or delay in surgery	5/8 2/8	[59, 63, 72, 79, 81] [60, 61]
	• Greater mortality with delay in surgery among patients with a Charlson comorbidity index (CCI) of 0 or 1 but improved survival for those with a CCI > = 3.	1/8	[82]
Non-operative management	Greater mortality with non-operative management	2/2	[54, 55]
Fracture type	Greater mortality with extra-capsular fractures	3/3	[51, 69, 70]
Perioperative fracture	Greater mortality with perioperative fractures	1/1	[81]
Local Factors			
Lower case-volume centers	Greater mortality with lower case-volume centers	2/2	[51, 73]
Poor nurse staffing	Greater mortality with poor nurse staffing	1/1	[73]
Inappropriate prescription	Greater mortality with inappropriate medication prescribing	1/1	[56]

## Discussion

This systematic review identified multiple predictors of poor functional outcomes and mortality for patients with hip fracture. Hand grip strength and frailty are two emerging predictors identified in this article. These two predictors were relatively new predictors identified in recent literature and were not found in the last major review [7]. Low hand grip strength was found to be a significant predictor of reduced gait speed and increased double support time [27]. Di Monaco M et al. reported a significant positive correlation between handgrip strength measured on admission to rehabilitation services and the Barthel Index scores assessed both on discharge from rehabilitation and at the 6-month follow-up [28]. The included studies analyzed hang grip strength as a continuous variable and did not specifically establish a threshold of absolute value above which the risk of poor functional outcome is higher. As for frailty, it is predictive of poorer basic ADL as well as 30-day mortality for hip fracture patients who underwent hip surgery [21, 52]. Krishnan M et al. reported that the 30-day mortality was 17.2% for patients of ‘high frailty’ (Frailty Index > 0.4), compared with 3.4% in ‘intermediate frailty’ patients (Frailty Index: 0.25–0.4) [58].

The above findings echoed with the emerging concept of “physical performance” as important functional capability measurement [11]. With an ageing population, frailty is becoming an important clinical syndrome resulting in poor functional outcomes, disability, and hospitalization [98, 99]. As there is increasing attention from researchers and policy makers on functional outcomes of patients, there is great interest in measuring and reporting them. However, various functional outcomes measures were used in the existing literature such as independence in mobility, FIM gain, Barthel Index efficacy, and EMS efficacy. Recent papers started to propose more specific and consistent methods to measure functional outcomes. For example, European Society for Clinical and Economic Aspects of Osteoporosis, Osteoarthritis and Musculoskeletal Diseases (ESCEO) working group on frailty and sarcopenia conducted comprehensive literature review and the experts panel recommended the use grip strength to measure muscle strength and 4-m gait speed or the Short Physical Performance Battery test to measure physical performance [11]. These recent developments would allow more standardized reporting of functional outcomes measured by validated, easy to use parameters in future medical literature.

The concept of physical performances has been changing over time. Previously, physical performances measures such as Timed-Get-Up-and-Go Test, Gait Speed Test and Modified Barthel Index were used as outcome measures under the domain of activity limitation [100]. ESCEO working group on frailty and sarcopenia now describes physical performance as a multidimensional concept where an objectively measured whole body function is related to the mobility of the individual [11]. In this recent review paper on the assessment of muscle function and physical performance in daily clinical practice by Charlotte Beaudart et al., a low grip strength is associated with poor outcomes and mortality [11]. Similarly, Robert D. Boutin et al. reported that CT findings of decreased thoracic paravertebral muscle size in older patients with hip fractures are associated with increased mortality [101]. While measurements of physical performance such as Gait Speed Test and Short Physical Performance Battery are strong predictors of loss of walking abilities and increased mortality, unfortunately such measurements may be biased in patients with hip fractures due to varying weight-bearing status.

This review found conflicting evidence for gender as a predictor of functional outcomes. Some studies reported that the female gender was a predictor of poorer functional outcomes as measured by ADLs [37] and EMS score [40]. Pajulammi HM et al. however concluded that the effect of gender on mobility recovery was minimal [25]. Kristensen MT et al. also reported that effect of gender on NMS was not significant [41]. However, female gender in other studies was found to be predictor of better functional outcomes as measured by early ambulation status [23], gait speed [27], and FIM gain [39, 45, 46]. This may be explained by the fact that the populations of these studies were heterogeneous. Future studies may focus on certain sub-populations to further elucidate the relationship between demographic factors and functional outcomes and mortality for patients with hip fracture.

With regard to the quality assessment of the included articles, the Newcastle-Ottawa Scale (NOS) was used to assess the quality of cohort studies. NOS covers three domains: selection of the cohorts, comparability of the cohorts, and assessment of the outcomes. Good quality studies are defined as those that achieve 3 or 4 stars in selection domain and 1 or 2 stars in comparability domain and 2 or 3 stars in outcome domain [16]. We used this scale because of it is easy to use and recommended by the Cochrane Collaboration [102, 103].

This review summarized and allows readers to have an oversight view of the predictors of poor functional outcomes and mortality for patients with hip fractures. Through identification of these predictors, healthcare providers would be better equipped to identify patients at risk of poor functional outcomes and/or death during their hospital admission. Healthcare providers can then tailor a patient-centered holistic care plan to assist patients to transit smoothly from the peri-operative period to the post-acute rehabilitation period. The post-acute care plan for these patients can also be tailored to facilitate better functional outcomes and lower mortality.

This paper has several limitations. Firstly, majority of the included articles were single-center observational studies, which are sensitive to selection bias and confounding factors. The number of good quality longitudinal cohort studies are sparse. Secondly, the measurements of the predictors are not standardized in different studies. For example, cognitive function is assessed by MMSE in most of the included studies but some used IQCODE, SPMSQ or cognitive FIM score. The inconsistencies in the instrument scales may have affected the sensitivity and specificity of the study in identifying the predictors. The search strategy of this article may also be further optimized by including more literature databases, non-English articles, and combining Mesh terms with free text keywords to further increase the comprehensiveness of the search strategy. Finally, the review protocol for this study was not registered.

## Conclusion

This systematic review identified multiple predictors of poor functional outcomes and mortality for patients with hip fracture. Hand grip strength and frailty are two emerging ones. These predictors would further inform healthcare providers of their patients’ health status and allow for early intervention for modifiable predictors.

## Supplementary information

**Additional file 1.** Search strategy and included/excluded articles.

## Data Availability

All data generated or analysed during this study are included in this published article and its supplementary information files.

## Abbreviations

ADL: Activities of Daily Living
ASA: American Society of Anesthesiologists
EMS: Elderly Mobility scale
FIM: Functional Independence Measure
IQCODE: Informant Questionnaire on Cognitive Decline in the Elderly
MMSE: Mini-Mental State Examination
NMS: New Mobility Score
ORs: Odds Ratios
POMA: Performance-Oriented Mobility Assessment
REFS: Reported Edmonton Frail Scale
SPMSQ: Short Portable Mental State Questionnaire
