# Supplementary material for: Predictors of poor functional outcomes and mortality in patients with hip fracture: a systematic review
Source: BMC Musculoskelet Disord. 2019 Nov 27;20:568. doi: 10.1186/s12891-019-2950-0 (PMC6882152; doi:10.1186/s12891-019-2950-0)
Supplement: Supplementary file 1 — Additional file 1. Search strategy and included/excluded articles. [file 12891_2019_2950_MOESM1_ESM.docx]

Search strategy for published literature in the past 15 years (from 1 Jan 2004 up to 30 May 2019) in three databases PubMed, EMBASE and Cochrane Library

Pubmed ((("Critical Care Outcomes"[Mesh] OR "Patient Outcome Assessment"[Mesh] OR "Outcome Assessment (Health Care)"[Mesh] OR "Patient Reported Outcome Measures"[Mesh] OR "Fatal Outcome"[Mesh] OR "Treatment Outcome"[Mesh])) AND "Hip Fractures"[Mesh]) AND predict* 322 articles

Embase (((((critical AND care AND outcomes OR patient) AND outcome AND assessment OR outcome) AND assessment AND health AND care OR patient) AND reported AND outcome AND measures OR fatal) AND outcome OR treatment) AND outcome AND hip AND fractures AND predict* 355 articles

Cochrane (Critical Care Outcomes OR Patient Outcome Assessment OR Outcome Assessment (Health Care) OR Patient Reported Outcome Measures OR Fatal Outcome OR Treatment Outcome) AND Hip Fractures AND predict* 1939 articles

Summary of the articles included for the systematic review

| **Reference Number** | **Study title** | **Authors** | **Study**  **Design** | **Region** | **Population Size** | **Study Setting** | **Factors studied** | **Outcomes measured** | **Findings** | **Level of Evidence of Study** |
| --- | --- | --- | --- | --- | --- | --- | --- | --- | --- | --- |
| 17 | Factors Influencing Outcomes of Older Adults After Undergoing Rehabilitation for Hip Fracture | McGilton KS et al.  (2016) | Cohort Prospective | Canada | 133 | Community Hospital | **Medical Factors**  - cognitive status - pre-fracture functional status | - Functional status  - Mobility | Cognitive impairment and poor pre-fracture functional impairment contribute to poor outcome. | Good |
| 18 | Prevalence of sarcopenia in acute hip fracture patients and its influence on short-term clinical outcome | González-Montalvo JI  et al.  (2016) | Cohort Prospective | Spain | 479 | Tertiary Hospital | **Medical Factors**  - sarcopenia  - grip strength | Functional status | Sarcopenia associated with worse functional prognosis at discharge. | Good |
| 19 | Influence of Cognitive Impairment on Mobility Recovery of Patients With Hip Fracture | Ariza-Vega P  et al.  (2017) | Cohort Prospective | Spain | 275 | Tertiary Hospital | **Medical Factor**  -cognitive status  **Socio-economic** -age **Surgical Factor**  -weight bearing status | Mobility recovery | Cognitive impairment, older age and non-weight-bearing status are negative prognostic factors for the recovery of mobility. | Good |
| 20 | Routine functional assessment for hip fracture patients | Pedersen TJ, Lauritsen JM  (2016) | Cohort Prospective | Denmark | 165 | Tertiary Hospital | **Medical Factor**  Pre-fracture functional status | - Independent walking ability  - Mortality | Pre-fracture functional status is a consistent predictor of outcomes. | Fair |
| 21 | Which frailty measure is a good predictor of early post-operative complications in elderly hip fracture patients? | Kua J et al.  (2016) | Cohort Prospective | Singapore | 144 | Tertiary Hospital | **Medical Factors**  Frailty scores  - MFC - REFS | - Postoperative complications  - BADL function level at six months | REFS is a good predictor of early postoperative complications and 6 months BADL function. | Fair |
| 22 | Self-Care and Mobility Following Post-acute Rehabilitation for Older Adults With Hip Fracture: A Multilevel Analysis. | Cary MP Jr  et al.  (2016) | Cohort Retrospective | United States | 35,264 | Multicenter | **Socio-economic** - age - race/ethnicity  **Medical Factors**  - cognitive status  - motor FIM  - comorbidities | BADL & Mobility at discharge | At the individual level, age, race/ethnicity, cognitive and motor FIM scores at admission, and tier comorbidities explained variance in self-care and mobility. | Good |

| 23 | Factors Associated With Early Functional Outcome After Hip Fracture Surgery | Cohn M.R. et al.  (2015) | Cohort Retrospective | United States | 99 | Tertiary Hospital | **Surgical Factors**  - delay to operation >48hrs - time of surgery  **Socio-economic**  - age & gender  **Medical Factor**  - ASA grade | Independent ambulation on postoperative day three | Delay to Surgery, higher ASA, males, older age associated with worse early functional outcomes. | Fair |
| --- | --- | --- | --- | --- | --- | --- | --- | --- | --- | --- |
| 24 | Factors Predicting Mobility and the Change in Activities of Daily Living After Hip Fracture: A 1-Year Prospective Cohort Study. | Mariconda M et al.  (2016) | Cohort Prospective | Italy | 552 | Multicenter  1 university & 2 community hospitals | **Medical Factors**  - pre-fracture functional status - comorbidities - cognitive status **Surgical Factor**  - non-weight bear status | - Ambulation ability  - Need for walking aids  - ADL status | Poor pre-fracture functional status, comorbidities, cognitive impairment, non-weight bear status are negative predictors. | Good |
| 25 | Pre- and perioperative predictors of changes in mobility and living arrangements after hip fracture--a population-based study | Pajulammi HM et al.  (2015) | Cohort Prospective | Finland | 1027 | Tertiary Hospital | **Socio-economic** - age & gender **Medical Factors**  - ASA grade - cognitive status - BMI - pre-fracture functional status **Surgical Factors**  - fracture type - delay to surgery | Changes in mobility & living arrangements | Older age, cognitive impairment, higher ASA, extra-capsular fractures, pre-fracture impaired mobility associated with decreased mobility.  Not living in own home independent risk factor for failure to regain pre-fracture mobility function. | Good |
| 26 | Prospective study of predictive factors of changes in pain and hip function after hip fracture among the elderly | Orive M  et al.  (2016) | Cohort Prospective | Spain | 740 | Multicenter  5 public hospitals | **Socio-economic** - Gender & age - socioeconomic status  **Medical Factor** - pre-fracture functional status,  **Surgical Factor** - delay to surgery | Pain and decline in function | Age ≥85, lower income, higher pre-fracture functional status, no referral to rehab upon discharge, delay to surgery are predictors of decline in function. | Good |
| 27 | Identification of gait domains and key gait variables following hip fracture | Thingstad P et al. (2015) | Cohort Prospective | Norway | 249 | Tertiary hospital | **Surgical Factor**  - fracture type **Medical Factors** - cognitive status  - grip strength  **Socio-economic** - Gender | Spatial and temporal gait variables | Cognitive decline, low grip strength, extra capsular fracture, handgrip strength and male gender were significant predictors of impaired gait. | Fair |
| 28 | Handgrip strength is an independent predictor of functional outcome in hip-fracture women: a prospective study with 6-month follow-up. | Di Monaco M et al.  (2015) | Cohort Prospective | Italy | 193 | Rehabilitation Hospital | **Medical Factor** Hand grip strength | Functional status using Barthel Index | Hand grip strength was significantly associated with the ability to function in ADL assessed both at the end of inpatient rehabilitation and at a 6-month follow-up in hip fracture women. | Fair |
| 29 | Factors Influencing Performance-Oriented Mobility After Hip Fracture | Martín-Martín LM  et al.  (2015) | Cohort Prospective | Spain | 186 | Tertiary hospital | **Socio-economic** - age  - length of stay  **Surgical Factor**  -fracture type **Medical Factors**  - pre-fracture functional status  - level of emotion distress | Performance-oriented mobility | Patients who are older, spend more days in hospital, have worse pre-fracture independence level or higher emotional distress levels at discharge, and sustain subtrochanteric or intertrochanteric fractures seem to have poorer performance-oriented mobility. | Fair |
| 30 | Adequate surgical treatment of periprosthetic femoral fractures following hip arthroplasty does not correlate with functional outcome and quality of life | Märdian S.  et al.  (2015) | Cohort Retrospective | Germany | 67 | Tertiary hospital | **Socio-economic** - age & gender **Medical Factors** - ASA  - comorbidities | Functional outcome and quality of life | Higher ASA & comorbidities influence functional outcome. | Poor |
| 31 | Factors associated with short-term functional recovery in elderly people with a hip fracture. Influence of cognitive impairment. | Uriz-Otano F et al.  (2015) | Cohort Prospective | Spain | 285 | Tertiary hospital | **Medical Factors** - cognitive status - pre-fracture walking ability | Functional outcomes  - ADL  - ability to walk | Pre-fracture walking ability and cognitive impairment are predictors of poor functional outcomes. | Fair |
| 32 | Determinants of outcome in hip fracture: role of daily living activities | Gialanella B et al.  (2015) | Cohort Prospective | Italy | 204 | Tertiary hospital | **Socio-economic** - age & gender **Surgical Factor**  - orthopedic treatment **Medical Factors**  - cognitive status - muscle strength | Post rehabilitation functional outcomes | Cognitive impairment is a major predictor of poor functional outcomes. | Fair |
| 33 | Postoperated hip fracture rehabilitation effectiveness and efficiency in a community hospital. | Tan AK et al.  (2014) | Cohort Retrospective | Singapore | 166 | Community hospital | **Socio-economic** - age & race **Medical Factors** - comorbidities - pre-fracture functional status - cognitive status | Rehabilitation effectiveness and efficiency &  Ambulation status | Dementia, multiple comorbidities, fewer rehabilitation sessions, Malay, older age, poor pre-fracture functional status associated with poor outcomes. | Fair |
| 34 | Non-weight-bearing status compromises the functional level up to 1 year after hip fracture surgery | Ariza-Vega P et al.  (2014) | Cohort Prospective | Spain | 194 | Tertiary hospital | **Socio-economic**  - age **Medical Factors** - ASA - cognitive status - pre-fracture functional status  **Surgical Factor**  - postoperative weight bearing status | Functional outcome  -FIM | Non-weight bear, older age, higher ASA, cognitive impairment, poor pre-fracture functional status: poor functional outcome. | Fair |
| 35 | Handgrip strength predicts persistent walking recovery after hip fracture surgery | Savino E  et al.  (2013) | Cohort Prospective | Italy | 504 | Multicenter  4 Tertiary hospitals | **Socio-economics** - age, gender  **Medical Factors**  - hand grip strength - comorbidities - cognitive status - serum vitamin D **Surgical Factors**  - type of fracture - time to surgery | Persistent walking recovery | After adjusting for confounders, hand grip strength was directly associated with persistent walking recovery. | Good |
| 36 | Outcomes and weight-bearing status during rehabilitation after arthroplasty for hip fractures. | Siebens HC et al.  (2012) | Cohort Prospective | United States | 224 | Multicenter  12 study sites | **Socio-economics** - age, gender  **Surgical Factor**  - weight bearing status | Cognitive, motor, and total FIM scores at discharge and at eight-month follow-up | WBAT had no bivariate association with cognitive or motor  function at discharge. | Good |
| 37 | Prognostic factors for self-rated function and perceived health in patient living at home three months after a hip fracture. | Sylliaas H  et al.  (2012) | Cohort Prospective | Norway | 490 | Multicenter  3 tertiary hospitals | **Medical Factors** - pre-fracture functional status  - cognitive status  **Socio-economic** - gender | Functional outcomes  - basic & instrument ADL | Pre-fracture use of mobility aid, cognitive impairment, female poor functional outcomes. | Good |

| 38 | Most patients regain pre-fracture basic mobility after hip fracture surgery in a fast-track programme | Kristensen M.T. et al.  (2012) | Cohort Prospective | Denmark | 213 | Tertiary hospital | **Socio-economic** - age **Medical Factor** - pre-fracture functional status, **Surgical Factor** - fracture type | Functional outcomes  -independency in mobility | Low pre-fracture NMS, intertrochanteric fracture more likely to not regain independency in mobility. | Fair |
| --- | --- | --- | --- | --- | --- | --- | --- | --- | --- | --- |
| 39 | Predictors of outcome following hip fracture rehabilitation | Semel J et al.  (2010) | Cohort Retrospective | United States | 557 | Tertiary hospital | **Socio-economic** - age  - gender - ethnicity - smoking & alcohol  - residence  **Medical Factors** - comorbidities  - pre-fracture functional status | FIM gain | Younger age, female, absence of diabetes, independent pre-fracture ambulation, not living alone before fracture, prescribed lesser medications predisposing to fall have better functional outcome. | Good |
| 40 | Relationship between admission albumin levels and rehabilitation outcomes in older patients | Luk JK et al.  (2011) | Cohort Retrospective | Hong Kong | 1604 | Multicenter  2 geriatric convalescent hospitals | **Medical Factors**  - Admission albumin - comorbidities - pre-fracture functional status **Socio-economic** - age & gender | Functional and motor gain using Barthel Index efficacy and EMS efficacy respectively | Female, older age, urinary incontinence negative predictors, Good pre-fracture functional status positive predictors. | Fair |
| 41 | Pre-fracture functional level evaluated by the New Mobility Score predicts in-hospital outcome after hip fracture surgery | Kristensen MT et al.  (2010) | Cohort Prospective | Denmark | 280 | Tertiary hospital | **Socio-economic** - age & gender **Medical Factors** - pre-fracture functional level  - mental status - health status **Surgical Factor**  - fracture type | Regain of independence of mobility during hospital stay | Older age, poor pre-fracture functional status, Intertrochanteric fractures more likely not to regain independence in mobility. | Good |
| 42 | Predictors of functional improvement among patients with hip fracture at a rehabilitation ward | Shakouri SK  et al.  (2009) | Cohort Prospective | Iran | 117 | Tertiary hospital | **Socio-economic** - age & gender  **Medical Factors**  - BMI - comorbidities **Surgical Factor**  - fracture type | Functional Independence Measure at discharge | Age, FIM score at admission to be independent predictors of FIM score at discharge. | Poor |

| 43 | Comorbid cognitive impairment and depression is a significant predictor of poor outcomes in hip fracture rehabilitation | Feng L et al.  (2010) | Cohort Prospective | Singapore | 146 | Community hospital | **Medical Factors** - Depression - cognitive status | Ambulatory status, modified Barthel Index, Quality of life | Depression and cognitive impairment predict poor functional outcomes. | Fair |
| --- | --- | --- | --- | --- | --- | --- | --- | --- | --- | --- |
| 44 | Factors predicting rehabilitation outcomes of elderly patients with hip fracture | Chin R et al.  (2008) | Cohort Prospective | Hong Kong | 303 | Multicenter  2 tertiary hospitals | **Medical Factors**  - comorbidities - premorbid mobility - cognitive status - admission FIM | Length of stay, placement, ambulation status, functional independence | Cognitive impairment and poor pre-fracture functional status predictor poor outcomes. | Fair |
| 45 | Rehabilitation outcome of hip fracture patients: the importance of a positive albumin gain | Mizrahi EH et al.  (2008) | Cohort Retrospective | Israel | 433 | Tertiary hospital | **Medical Factors** - Albumin gain - Cognitive status - pre-fracture functional status - Comorbidities  **Socio-economics** - age, gender | Functional outcomes  -FIM at discharge | Higher MMSE score at admission, female, positive albumin gain, and good pre-fracture functional status predict higher FIM score at discharge. | Fair |
| 46 | Admission albumin levels and functional outcome of elderly hip fracture patients: is it that important? | Mizrahi EH et al.  (2007) | Cohort Retrospective | Israel | 449 | Tertiary hospital | **Medical Factors** - Admission albumin level - Cognitive status  - pre-fracture functional status - comorbidities **Socio-economics** - age, gender | Functional outcomes  -FIM at discharge | Higher MMSE score at admission, female, good pre-fracture functional status predict higher FIM score at discharge.  Albumin level did not. | Fair |
| 47 | The effect of co-morbidity on the rehabilitation process in elderly patients after hip fracture | Press Y et al. (2007) | Cohort Prospective | Israel | 102 | Tertiary hospital | **Medical Factors** - FIM at admission  - cognitive status  - comorbidities | Functional outcomes  -FIM at discharge | Cognitive impairment and Comorbidities predict poor outcomes. | Fair |
| 48 | In Hospital and 3-Month Mortality and Functional Recovery Rate in Patients Treated for Hip Fracture by a Multidisciplinary Team | Rostagno C et al.  (2016) | Cohort Prospective | Italy | 121 | Tertiary hospital | **Socio-economics** - age, gender **Medical Factors**  - comorbidities  - preserved basic ADL >3 - mobility index  **Surgical Factors**  - time to surgery  - fracture type | Three-month mortality | Age, Dementia, >two comorbidities, loss of >three basic ADL are significant predictors. | Fair |
| 49 | Association of nutritional status as measured by the Mini-Nutritional Assessment Short Form with changes in mobility, institutionalization and death after hip fracture | Nuotio M et al.  (2016) | Cohort Prospective | Finland | 472 | Tertiary hospital | **Socio-economic** - age & gender - living arrangements **Medical Factors** - ASA - cognitive status - mobility level - Mini-Nutritional Assessment Short Form score | Four-month mortality | Malnourished, higher age, male gender, higher ASA grade, non-independent mobility level and not living in own home were significant predictors of four-month mortality. | Good |
| 50 | Short-term outcomes following hip fractures in patients at least 100 years old | Manoli A  et al.  (2017) | Cohort Retrospective | United States | 168087 | Multicenter  Via New York SPARCS | **Socio-economic**  - age & gender  - discharge location **Medical Factor**  - comorbidities **Surgical Factors**  - time to surgery - fracture types | In-hospital mortality | Increasing age, Male gender, increasing comorbidities predict in-hospital mortality. | Good |

| 51 | Comparing the contributions of acute and post-acute care facility characteristics to outcomes after hospitalization for hip fracture | Neuman M.D et al.  (2017) | Cohort Retrospective | United states | 45,996 | Multicenter  Tertiary hospitals and nursing homes | **Socio-economic**  - nursing home characteristics - age & gender - Acute and post-acute Care Facility Factors  **Medical Factor** - comorbidities  **Surgical Factor**  - fracture type | Mortality time frame  - in-hospital mortality  - 30-day mortality  - 180-day mortality | Older age, male, fracture types, increasing comorbidities, subtrochanteric fracture, low volume center higher inpatient mortality risk.  Nursing home factors explained three times more variation in the odds of 30-day mortality, seven times more variation in the odds of 180-day mortality and eight times more variation in the odds of 180-day death. | Good |
| --- | --- | --- | --- | --- | --- | --- | --- | --- | --- | --- |
| 52 | Impact of frailty on outcomes in geriatric femoral neck fracture management: An analysis of national surgical quality improvement program dataset | Dayama A et al.  (2016) | Cohort Retrospective | United States | 3121 | Multicenter  Tertiary hospitals | **Medical Factors** - Frailty - comorbidities  - ASA grade **Socio-economic**  - age | 30-day mortality | Frailty is an independent predictor of mortality. Older age, higher ASA class, increasing comorbidities risk factors. | Good |
| 53 | Risk factor profiles for early and delayed mortality after hip fracture: Analyses of linked Australian Department of Veterans' Affairs databases | Ireland AW  et al.  (2015) | Cohort Retrospective | Australia | 2552 | Multicenter  Public & private hospitals, Residential Aged Care(RAC) | **Socio-economic** - age & gender  - pre-fracture  accommodation **Medical Factors**  - comorbidities - cancer - cardiac & renal failure, cerebrovascular  Disease  **Surgical Factors**  - fracture type - treatment options | Mortality at intervals  from 30 days to four years | Risk of death within one year was increased  by male gender, increasing age, pre-fracture RAC residency, transfer to intensive care and coexistent cancer,  cardiac and renal failure, cerebrovascular disease and pressure ulcers. Patients selected for rehabilitation  had lower mortality rates. | Good |

| 54 | Survival and functional outcomes after hip fracture among nursing home residents. | Neuman MD et al.  (2014) | Cohort Retrospective | United states | 60111 | Multicenter  Nursing homes | **Socio-economic** - age, gender & race  **Medical Factors** - comorbidities - cognitive status **Surgical Factor**  treatment options | 180-day mortality | Older age, non-operative fracture management, advanced comorbidity, advanced cognitive impairment greatest decreases in survival. | Good |
| --- | --- | --- | --- | --- | --- | --- | --- | --- | --- | --- |
| 55 | Can preoperative scoring systems be applied to Asian hip fracture populations? Validation of the Nottingham Hip Fracture Score (NHFS) and identification of preoperative risk factors in hip fractures | Kau C.Y., Kwek E.B  (2014) | Cohort Prospective | Singapore | 212 | Tertiary hospital | **Socio-economic** - age, gender, race  **Medical Factors** - comorbidities - pre-fracture functional status  - admission haemoglobin & Abbreviated Mental Test **Surgical Factors**  - days to surgery  - fracture type | One-month and one-year mortality | Surgically treated patients have lower odds ratio for mortality up to one year. | Fair |
| 56 | Inappropriate prescribing as a predictor for long-term mortality after hip fracture | Gosch M. et al.  (2014) | Cohort Retrospective | Austria | 457 | Tertiary hospital | **Medical Factors**  -STOPP and START criteria to assess the appropriateness of medication prescribing -comorbidity - functional status  **Socio-economic**  - age | all-cause mortality rate at three years | Non-survivors were significantly older, had more comorbidities, were less functional and received a higher number of prescriptions. | Fair |
| 57 | Risk factors for complications and in-hospital mortality following hip fractures: A study using the National Trauma Data Bank | Belmont Jr. P.J et al.  (2014) | Cohort Retrospective | United States | 44,419 | Multicenter  90 national medical centers | **Socio-economic**  - Patient demographics **Medical Factors**  - comorbidities - cardiac disease  - renal disease - injury-specific factors | In-patient mortality | Dialysis, presenting in shock, cardiac disease, male gender were significant predictors of mortality. | Good |
| 58 | Predicting outcome after hip fracture: Using a frailty index to integrate comprehensive geriatric assessment results | Krishnan M et al.  (2014) | Cohort Prospective | United Kingdom | 178 | Tertiary hospital | **Medical Factor** Frailty Index | 30-day mortality | Frailty index is a better predictor of mortality. | Fair |

| 59 | Surgical treatment of hip fractures: Factors influencing mortality | Dailiana Z.H. et al.  (2013) | Cohort Prospective | Greece | 218 | Tertiary hospital | **Socio-economic**  -Demographic characteristics  - pre-fracture residence  **Medical Factors** - pre-fracture functional status - comorbidities,  **Surgical Factors**  - fracture type - time to surgery | Mortality time frame  - in-hospital mortality  - 30-day mortality  - One-year mortality | Surgical delay >48 hours, >three comorbidities, male gender, and advanced age increased the risk of in-hospital and one-year mortality. | Fair |
| --- | --- | --- | --- | --- | --- | --- | --- | --- | --- | --- |
| 60 | Surgical Time of Day Does Not Affect Outcome Following Hip Fracture Fixation | Switzer J.A. et al.  (2013) | Cohort Retrospective | United States | 859 | Level 1 Trauma Center | **Medical Factors** - comorbidities - ASA score **Surgical Factors** - time to surgery - procedure length, - total time in the surgery - intraoperative fracture | 30-day mortality | No difference in 30-day mortality based on the time of day the surgery. | Good |
| 61 | Time to surgery and 30-day morbidity and mortality of periprosthetic hip fractures. | Griffiths EJ et al.  (2013) | Cohort Retrospective | United States | 60 | Tertiary hospital | **Medical Factors** - ASA grade - Cognitive status  **Surgical Factor**  - delay to surgery>72hrs | 30-day mortality | Statistically not significant for mortality rate associated with delay to surgery >72hrs. | Poor |
| 62 | Undisplaced intracapsular hip fractures in the elderly: Predicting fixation failure and mortality. A prospective study of 162 patients | Clement N.D. et al.  (2013) | Cohort Prospective | United Kingdom | 162 | Tertiary hospital | **Socio-economic**  - Patient demographics  **Medical Factor**  - ASA grade | One-year mortality | High ASA grade significant independent predictor of one-year mortality. | Fair |

| 63 | Early and ultra-early surgery in hip fracture patients improves survival | Uzoigwe C.E. et al.  (2013) | Cohort Retrospective | United Kingdom | 2056 | Multicenter  England and Wales National Hip Fracture Data Base | **Socio-economic**  - age & gender - residence prior to admission **Medical Factor**  - ASA grade **Surgical Factor**  - time to surgery | In-hospital mortality | Older age, male, high ASA grade, delay of surgery >48hrs associated increased risk of in-hospital mortality.  Ultra-early surgery (within 12 h) or early  surgery (within 24h), conferred a significant survival advantage. | Good |
| --- | --- | --- | --- | --- | --- | --- | --- | --- | --- | --- |
| 64 | Older people with hip fracture and IADL disability require earlier surgery | Pioli G. et al.  (2012) | Cohort Prospective | Italy | 806 | Multicenter  3 tertiary hospitals | **Socio-economic**  - age, gender, living arrangements **Medical Factors**  - cognitive status  - comorbidity - pre-fracture functional status **Surgical Factors**  - fracture type  - time to surgery | One-year mortality | Surgery delay is a strong independent factor for one-year mortality in the frail older people with pre-fracture functional impairment. | Good |
| 65 | Predictors of 5 year survival following hip fracture | Stewart N.A. et al.  (2011) | Cohort Prospective | United Kingdom | 2640 | Tertiary hospital | **Socio-economic**  - age  - residential status **Medical Factors**  - cognitive status  - mobility | Five-year mortality | Increased survival was shown for the following variables:  - age < 80 years - AMT $\geq$ 7/10  - independent mobility - admitted from own home | Good |
| 66 | Risk factors for in-hospital post-hip fracture mortality | Frost S.A  et al.  (2011) | Cohort Prospective | Australia | 1504 | Tertiary hospital | **Socio-economic**  - age, & gender  **Medical Factors** - comorbidities - heart disease  - liver disease  **Surgical Factor**  - fracture type | In-hospital mortality | Advancing age, male and pre-existing concomitant diseases such as congestive heart failure and liver disease were the main risk factors for in-hospital mortality. | Good |

| 67 | Improved Survival of Hip Fracture Patients Treated Within a Comprehensive Geriatric Hip Fracture Unit, Compared With Standard of Care Treatment | Adunsky A et al.  (2011) | Cohort Retrospective | Israel | 3114 | Tertiary hospital | **Socio-economic**  - age & gender **Medical Factors**  - concomitant medical conditions  **Surgical Factor**  - time to surgery  **Others** - model of care | Mortality time frame  - 30-day mortality  - 90-day mortality  - one-year mortality | Male, older age,  Diabetes, and number of operations were predictive of increased one-year mortality.  Crude and adjusted mortality rates are  lower in a geriatric hip fracture unit, as compared with the common standard of care model. | Good |
| --- | --- | --- | --- | --- | --- | --- | --- | --- | --- | --- |
| 68 | Clinical and biochemical prediction of early fatal outcome following hip fracture in the elderly | Talsnes O et al.  (2011) | Cohort Prospective | Norway | 302 | Multicenter  2 tertiary hospitals | **Socio-economic**  - age & gender  **Medical Factors**  - ASA grade - Creatine Kinase - myocardium specific Creatine kinase - Troponin T | Three-month postoperative mortality | Age, male, higher ASA grade correlate with mortality  High Troponin T >0.04 μg/l correlated significantly with death. | Good |
| 69 | Factors affecting mortality of frail hip-fractured elderly patients | Hershkovitz A et al.  (2010) | Cohort Prospective | Israel | 376 | Rehab center | **Socio-economic**  - age & gender **Surgical Factors**  - fracture type - treatment options - time to surgery **Medical Factors** - comorbidities | Two-year mortality | Dementia and age  were independent predictors of mortality. | Good |
| 70 | Factors at admission associated with 4 months outcome in elderly patients with hip fracture. | Björkelund KB et al.  (2009) | Cohort Prospective | Sweden | 428 | Tertiary hospital | **Socio-economic**  - Demographics  - place of residence **Medical Factors** - functional Status - cognitive status  - walking ability  - ASA grade  **Surgical Factors**  - fracture type & treatment | Four-month mortality | Four-month mortality associated with age $\geq$85, ASA grade3&4, male, more extensive fractures, dependency in living, dementia, Haemoglobin <10g/dL, Creatinine > 100 mmol/L, inability to walk alone. | Good |

| 71 | Predictive value of six risk scores for outcome after surgical repair of hip fracture in elderly patients | Burgos E  et al. (2008) | Cohort Prospective | Spain | 232 | Tertiary hospital | **Medical Factors** - ASA grade - Barthel Index - Goldman Index  - POSSUM index  - Charlson Index - Visual Analogue Scale for Risk (RISK-VAS) scale | 90-day mortality | None of the scales proved capable of predicting 90-day mortality. | Fair |
| --- | --- | --- | --- | --- | --- | --- | --- | --- | --- | --- |
| 72 | Risk factors for mortality in geriatric hip fractures: a compressional study of different surgical procedures in 785 consecutive patients. | Ercin E et al.  (2017) | Cohort Retrospective | Turkey | 785 | Multicenter  Tertiary hospitals | **Socio-economic**  - age,& gender  **Surgical Factors**  - type of fracture - type of surgery  - time to surgery  - operation length  **Medical Factors**  - ASA grade - comorbidities - anesthesia type - blood transfusion requirement  - intensive care unit requirement | Two-year mortality | Transfusion requirement during the hospital stay, ASA grade four and having more than two comorbidities are found to be significant  risk factors for mortality. | Good |

| 73 | Hospital Characteristics, Inpatient Processes of Care, and Readmissions of Older Adults with Hip Fractures | Elkassabany N.M et al.  (2016) | Cohort Retrospective | United States | 458,526 | Multicenter  3,485 acute care hospitals | **Local Factors**  Information on hospital case volumes, teaching status, bed count, nurse staffing, and technological capabilities from Medicare files | 30-day mortality | Better nurse staffing and higher case volumes are associated with lower rates of mortality. | Good |
| --- | --- | --- | --- | --- | --- | --- | --- | --- | --- | --- |
| 74 | Short-term complications in hip fracture surgery using spinal versus general anaesthesia. | Fields AC et al.  (2015) | Cohort Retrospective | United States | 6133 | Multicenter  >200 hospitals | **Surgical Factor**  - method of anaesthesia | 30-day morbidity and mortality | No difference in  mortality in patients who undergo hip fracture repair with spinal versus general anaesthesia. | Good |
| 75 | Does early functional outcome predict 1-year mortality in elderly patients with hip fracture? | Dubljanin-Raspopović E et al. (2013) | Cohort Prospective | Serbia | 288 | Tertiary hospital | **Socio-economic**  - age & gender **Medical Factors**  - cognitive status  - ASA grade - pre-fracture functional level  - functional  level at admission & discharge  **Surgical Factor**  - time to surgery | 1-year mortality | Older age, high ASA grade, cognitive impairment, poor pre-fracture functional status related to lower discharge motor FIM score  In multivariate analysis, FIM at discharge predictor of mortality.  In univariate analysis, age, pre-fracture motor FIM, FIM at discharge, delirium are predictors of mortality. | Good |
| 76 | Prediction of survival, second fracture, and functional recovery following the first hip fracture surgery in elderly patients | Kim SM et al.  (2012) | Cohort Prospective | Korea | 415 | Tertiary hospital | **Socio-economic**  - age, residence place  **Medical Factors** - previous fracture  - preoperative ambulatory ability  - comorbidities - ASA grade - cognitive status | Two-year functional outcomes and mortality | Advanced age, cancer, a prior fracture history, and a solitary life were found to be significantly associated with the risk of increased two-year mortality.  Malignancy and cognitive impairment associated with a poor functional outcome. | Good |

| 77 | Use of early indicators in rehabilitation process to predict one-year mortality in elderly hip fracture patients | Dubljanin-Raspopović E et al. (2012) | Cohort Prospective | Serbia | 344 | Tertiary hospital | **Socio-economic**  - Age & gender  - residence place  **Medical Factors** - ASA grade - Pre-fracture functional status,  - cognitive status | Ambulation status at discharge  one-year mortality | Older age, severe cognitive impairment, lower functional level before injury, postoperative delirium and pressure ulcers have higher chance of not recovering their gait ability at discharge, and being dead one year after hip fracture. | Good |
| --- | --- | --- | --- | --- | --- | --- | --- | --- | --- | --- |
| 78 | Is pre-fracture functional status better than cognitive level in predicting short-term outcome of elderly hip fracture patients? | Dubljanin-Raspopović E et al. (2012) | Cohort Prospective | Serbia | 337 | Tertiary hospital | **Medical Factors**  - Cognitive status  - Pre-fracture functional status | Four-month functional outcomes and mortality | Cognitive status and pre-fracture motor FIM predict mortality at four months  Pre-fracture ADL predict absolute motor FIM gain at four months. | Good |
| 79 | A multicenter survey on profile of care for hip fracture: Predictors of mortality and disability | Maggi S  et al. (2010) | Cohort Prospective | Italy | 3707 | Multicenter  9 tertiary hospitals | **Socio-economic**  - Age & gender  - residence place  - date and destination at discharge  **Medical Factors**  - pre-fracture walking ability - walking aid  - ASA grade **Surgical Factors**  - type of fracture - time to surgery - type of surgery | Six-month functional status and mortality | Six-month mortality was positively associated with increasing age, comorbidity, pre-fracture functional disability, and delay to surgery > 48 h.  Walking disability was associated with older age, comorbidity, disability before fracture, delay to surgery > 24hrs.  Higher levels of functional status at six months were independently associated with surgery occurring within 24h. | Good |
| 80 | Effect of inpatient quality of care on functional outcomes in patients with hip fracture | Siu AL et al. (2006) | Cohort Prospective | United States | 554 | Multicenter  Four tertiary hospitals | **Others**  - process of care (e.g. mobilization after surgery and timely physical therapy) | Six-month survival and  FIM locomotion/self care/transferring at 2 and 6 months | Better process of care was associated with improvement on all 3 scales of function (locomotion, self-care and transferring at 2 months but was not associated with 6-month survival | Good |
| 81 | Factors affecting postoperative mortality of patients with displaced femoral neck fracture | Petersen MB et al. (2006) | Cohort  Retrospective | Denmark | 1186 | Tertiary hospital | **Socio-economic**  - Age & gender  **Medical Factors**  - cardiac arrest (perioperatively)  - Cardiac arrhythmia (perioperatively)  - heart failure (postoperatively)  - dementia  - stroke (postoperatively) **Surgical Factors**  - time to surgery  - duration of surgery  - number of reoperations  - operation time of day  - luxation of prothesis (perioperatively)  - perioperative fracture (operatively treated)  - bleeding during surgery  - blood transfusion  **Others**  - Length of orthopaedic hospital stay | Mortality at 3 months following hemiarthroplasty | Cardiac complications, dementia, male sex, older age, time to surgery, stroke and dislocation of the prosthesis and perioperative fracture negatively inﬂuence mortality at 3 months. | Good |
| 82 | Factors inﬂuencing survival following hip fracture among octogenarians and nonagenarians in the United States | Bokshan SL et al. (2018) | Cohort  Retrospective | United States | 284 | Tertiary trauma center | **Medical Factors** - comorbidities (Charlson Comorbidity Index)  **Surgical factors**  - surgical delay | Mortality at 1 year and 5 years post operation | Octogenarians with a preoperative CCI>=3 had an increased mortality at both 1 year and 5 years postoperatively. Nonagenarians with a preoperative CCI>= 3 had an increased mortality at 1 year but not 5 years.  A surgical delay of 48 h resulted in a signiﬁcant increased postoperative mortality among patients with a CCI of 0 or 1 but improved survival for patients with a CCI >= 3. | Good |
| 83 | Functional outcomes and mortality vary among different types of hip fractures: A function of patient characteristics | Cornwall R et al. (2004) | Cohort Prospective | United States | 537 | Multicenter  Four tertiary hospitals | **Socio-economic**  - Age  **Medical Factors** - preinjury function  **Surgical factors**  - types of hip fractures | Mortality at 6 months  Functional outcome at 6 months | Preinjury function (FIM score) as an independent predictor of mortality at 6 months.  Patient age and preinjury functions are independent predictors of functional outcome (FIM score) at 6 months. | Good |
| 84 | Functional Recovery Among Elderly People One Year After Hip Fracture Surgery | Lin PC and Chang SY (2004) | Cohort Prospective | Taiwan | 103 | Tertiary hospital | **Socio-economic**  - age, marital status, gender, residence  **Medical Factors** - ADL, instrumental ADL before fracture,  - eyesight, hearing ability, walking status, use of walking aid and history of falling down  - dis- ease and medication history | ADL and instrumental ADL recovery within one year after hip fracture | Ability to walk outdoors pre-fracture is the significant predictor for ADL recovery within one year after hip fracture.  For instrumental ADL, significant predictors included doing housework, marital status and use of walking aids before fracture. | Good |
| 85 | Handgrip strength but not appendicular lean mass is an independent predictor of functional outcome in hip-fracture women: a short-term prospective study | Monaco MD (2014) | Cohort Prospective | Italy | 123 | Rehabilitation hospital | **Medical Factors**  - appendicular lean mass  - grip strength at the nondominant arm | ADL by using the Barthel Index, and lower limb performance by using the Timed Up and Go test at the end of inpatient rehabilitation | Grip strength signiﬁcantly predicted short-term functional outcome in women after a hip fracture. | Fair |
| 86 | Inpatient Rehabilitation Outcome After Hip Fracture Surgery in Elderly Patients: A Prospective Cohort Study of 946 Patients | Lieberman D et al. (2006) | Cohort Prospective | Israel | 946 | Rehabilitation center | **Socio-economic**  - age,  **Medical Factors**  - comorbidity including smoking, hemoglobin level, serum albumin, thyroid-stimulating hormone, folic acid, and vitamin B12, pre-fracture FIM, Folstein Mini-Mental State Examination, Geriatric Depression Scale | FIM score at the end of rehabilitation | Prefracture FIM scale, serum albumin at discharge, Folstein Mini-Mental State Examination, visual impairment, dyspnea at mild exertion (New York Heart Association class III), age, post-stroke motor impairment, and decreased serum folic acid were signiﬁcantly and independently associated with rehabilitation outcome. | Good |
| 87 | Native hip dislocation at acetabular fracture predicts poor long-term outcome | Nicholson JA et al. (2018) | Cohort  Retrospective | United Kingdom | 480 | Tertiary hospital | **Surgical Factors**  - presence of associated posterior hip dislocation with hip fracture | Oxford Hip Score and SF-12 at long term | Acetabular fractures with an associated dislocation have worse long-term functional outcomes compared to acetabular fractures without a dislocation. | Good |
| 88 | Patient survival and surgical re-intervention predictors for intracapsular hip fractures | Quevedo DG et al. (2017) | Cohort  Retrospective | Spain | 356 | Tertiary hospital | **Medical Factors**  - age adjusted CCI and ASA score | Mortality at 2 years after hip surgery | Both age adjusted CCI and ASA scales were able to predict the 2-year survival of patients with intracapsular hip fractures. | Good |
| 89 | Predicting Outcomes after Hip Fracture Repair | Kagaya H et al. (2005) | Cohort Prospective | Japan | 63 | Tertiary hospital | **Socio-economic**  - age  **-** living status (alone or with someone, and at own or at a nursing home)  **Medical Factors**  - FIM subscales  - dementia  - heart diseases  - stroke  **Surgical factors**  **-** type of fracture  **Others**  **-** length of stay in hospital | Independent walking at discharge and at 6-month follow-up | Mobility and social cognition FIM subscales pre-fracture were signiﬁcantly related to independent walking both at discharge and at 6 months follow-up | Good |
| 90 | Predictors for rehabilitation outcome in Asian geriatric hip fracture patients | Gatot C et al, (2016) | Cohort Prospective | Singapore | 153 | Tertiary hospital | **Socio-economic**  - age, gender  **Medical Factors**  **-** comorbidities  **Surgical factors**  - Fracture type | RFG at one year after surgery | Age 80–89 years was a predictor for poor RFG, and hypercholesterolaemia was a predictor for good RFG. | Good |
| 91 | Predictors of a Change and Correlation in Activities of Daily Living after Hip Fracture in Elderly Patients in a Community Hospital in Poland: A Six-Month Prospective Cohort Study | Ganczak et al. (2018) | Cohort Prospective | Poland | 120 | Tertiary hospital | **Socio-economic**  - age, gender, residence (urban/rural), **Medical factors**  **-** cognitive status  - CCI index  - ASA class  **Surgical factors**  **-** type of fracture  - time to surgery  - type of anesthesia (local/general),  - surgical type (osteosynthesis/arthroplasty)  - surgical treatment duration (minutes); - time to mobilization  **Others:**  - length of hospital stay  - post-discharge rehabilitation | Katz ADL index and Lawton-Brody IADL scale at 6 months follow up | Regaining ADL after six months was more likely in patients with pre-fracture intact intellectual function and independence in pre-fracture ADL; Regaining instrumental ADL was more likely in younger patients and those with higher pre-fracture instrumental ADL scores. | Good |
| 92 | Predictors of functional outcome following femoral neck fractures treated with an arthroplasty: limitations of the Harris hip score | Reuling EMBP et al. (2012) | Cohort Prospective | The Netherlands | 252 | Multicenter  one academic and seven district hospitals | **Socio-economic**  - age,  **Medical factors**  - pre-operative comorbidity, ASA-classification, type of arthroplasty  **Surgical factors**  **-** surgeon (resident or consultant),  - interval between trauma and operation,  - blood loss,  - peri-and post-operative inhospital complications  - general post-operative inhospital complications | Harris hip score at one year and 5 years follow up | Age and the existence of pre-operative co-morbidities appeared to be predictors of the functional outcome after 1 and 5 years. | Jadad score 3 |
| 93 | Predictors of long-term survival after hip fractures?—5-year results of a prospective study in Germany | Knauf T et al. (2019) | Cohort Prospective | Germany | 395 | Tertiary hospital | **Socio-economic**  - age, gender, residential status,  **Medical factors**  **-** Mini-Mental State Examination (MMSE), pre-fracture Barthel Index, Charlson Comorbidity Score, ASA Score, EQ5D . | Mortality at 5 years | Male gender, higher age, lower Barthel Index, lower Charlson Comorbidity Score, lower Mini-Mental State Examination, and delirium during hospitalization are risk factors for long term mortality | Good |
| 94 | The effect of osteoporotic treatment on the functional outcome, re-fracture rate, quality of life and mortality in patients with hip fractures: A prospective functional and clinical outcome study on 520 patients | Makridis KG et al. (2015) | Cohort Prospective | Greece | 520 | Multicenter  Regional hospitals | **Socio-economic**  - living conditions  **Medical factors**  - osteoporotic treatment, mental status, rehabilitation  **Surgical factors**  - surgery related parameters, type of fracture | Functional outcome (e.g. Parker mobility score Walking speed test Time up and go test) and mortality at 2 years | Osteoporotic treatment proved to be an important predictor of functional recovery. | Good |
| 95 | The Effect of Perioperative Anemia on Clinical and Functional Outcomes in Patients With Hip Fracture | Halm EA et al. (2004) | Cohort Prospective | United States | 550 | Multicenter  Tertiary hospitals | **Medical factors**  - preoperative and postoperative hemoglobin level | Deaths and Functional Independence Motor mobility scores within 60 days of discharge | Higher hemoglobin levels on admission were associated with lower mortality. | Good |
| 96 | The Influence of Cognitive Function on Outcome After a Hip Fracture | Söderqvist A et al. (2006) | Cohort Prospective | Sweden | 213 | Tertiary hospital | **Socio-economic**  **-** age, gender  **Medical factors**  - cognitive function measured by Short Portable Mental Status Questionnaire | Charnley hip score, activities of daily living status, and mortality. | A Short Portable Mental Status Questionnaire score of <3 and male gender were associated with an increased mortality rate during the first twelve months. Patients with a score of <3 had a significantly worse outcome with regard to the ability to walk and to perform the activities of daily living, at the time of the final follow- up | Good |
| 97 | The influence of pre-existing radiographic osteoarthritis on functional outcome after trochanteric fracture | Boese CK et al. (2015) | Cohort Prospective | Germany | 188 | Tertiary hospital | **Socio-economic**  -age, gender.  **Medical factors**  - pre-existing radiographic osteoarthritis  - ASA physical status score, MMSE on admis- sion and pre-fracture Barthel Index | Harris hip score, the timed up and go test and the Barthel Index at six and 12 months | There is a significant association between the grade of osteoarthritis and the Harris hip score at six months postoperatively | Good |

**Abbreviations:**

ADL: Activities of Daily Living

ASA: American Society of Anesthesiologists

BMI: Body Mass Index

CCI: Charlson Comorbidity Index

EMS: Elderly Mobility Scale

FIM: Functional Independence Measure

MFC: Modified Fried Criteria

POSSUM: Physiological and Operative Severity Score for the enUmeration of Mortality and Morbidity

REFS: Reported Edmonton Frail Scale

RFG: Relative Functional Gain

STOPP: Screening Tool of Older Person's Prescriptions

START: Screening Tool to Alert doctors to Right Treatment

SPARCS: Statewide Planning and Research Cooperative System

WBAT: weight bear as tolerated

Excluded articles after full text review (publication titiles):

1. Reported outcomes not related to study outcomes (n=20)

Polypharmacy and adverse outcomes after hip fracture surgery

Depression Predicts Functional Outcome in Geriatric Inpatient Rehabilitation

Perioperative risk factors in patients with a femoral neck fracture - influence of 25-hydroxyvitamin D and C-reactive protein on postoperative medical complications and 1-year mortality

Short-term complications in hip fracture surgery using spinal versus general anaesthesia.

Serum levels of 25-hydroxyvitamin D and functional outcome among postmenopausal women with hip fracture

Dependence for basic and instrumental activities of daily living after hip fractures.

Predictors of change in 'discharge destination' following treatment for fracture neck of femur.

Predictors of length of hospital stay in elderly hip fracture patients.

Relation between prefracture characteristics and perioperative complications in the elderly adult patient with hip fracture.

Rehabilitation outcomes of older patients at 6 months follow-up after discharged from a geriatric day hospital (GDH).

Race/ethnicity and outcomes following inpatient rehabilitation for hip fracture

Prospective study of predictive factors of changes in pain and hip function after hip fracture among the elderly.

Outcome following proximal femoral fracture in Northern Ireland

Effect of inpatient quality of care on functional outcomes in patients with hip fracture

Decreases in heart rate variability are associated with postoperative complications in hip fracturepatients

Predictors of Functional Recovery Following Periprosthetic Distal Femur Fractures

Type 2 Diabetes and Risk of Hip Fractures and Non-Skeletal Fall Injuries in the Elderly: A Study From the Fractures and Fall Injuries in the Elderly Cohort (FRAILCO).

Twelve-month work-related outcomes following hip fracture in patients under 65 years of age.

Heterogeneity in hip fracture patients: age, functional status, and comorbidity.

Predictors of early failure in young patients with displaced femoral neck fractures.

1. Inappropriate Methodology (n=8)

Co-morbidities in elderly patients with hip fracture: Recommendations of the ISFR-IOF hip fracture outcomes working group

The influence of cognitive function on outcome after a hip fracture

Preoperative prediction of early physical function in elder patients undergoing hip arthroplasty using a subjective physical activity questionnaire

A Systematic Review and Meta-analysis Examining the Impact of Incident Postoperative Delirium on Mortality

Timing matters in hip fracture surgery: patients operated within 48 hours have better outcomes. A meta-analysis and meta-regression of over 190,000 patients

Outcomes following hip fracture surgery: a 2-year prospective study

Mild to moderate cognitive impairment is a major risk factor for mortality and nursing home admission in the first year after hip fracture

The impact of surgeon volume and hospital volume on postoperative mortality and morbidity after hip fractures: A systematic review

1. Poster Abstract (n=4)

Home-based post-acute care for older patients with hip fracture in Taiwan: A prospective cohort study

Short and medium term mortality in elderly patients with hip fracture: A prospective study

Functional outcome and short-term mortality after surgery for hip fractures

Hip Fracture and the Weekend Effect: Does Weekend Admission Affect Patient Outcomes?
